# Association of human myxovirus resistance protein A with severity of COVID-19

**DOI:** 10.1186/s12879-022-07753-0

**Published:** 2022-09-28

**Authors:** Otto Lehtinen, Niklas Broman, Matti Waris, Tytti Vuorinen, Ville Peltola, Eliisa Löyttyniemi, Jarmo Oksi, Thijs Feuth

**Affiliations:** 1 Department of Pulmonary Diseases and Clinical Allergology, Turku University Hospital and University of Turku, Turku, Finland; 2 Department of Infectious Diseases, Turku University Hospital and University of Turku, Turku, Finland; 3 Department of Clinical Microbiology, Turku University Hospital and University of Turku, Turku, Finland; 4 Department of Paediatrics and Adolescent Medicine, Turku University Hospital and University of Turku, Turku, Finland; 5 Department of Biostatistics, University of Turku, Turku, Finland

**Keywords:** COVID-19, SARS-CoV-2, Human myxovirus resistance protein A, MxA

## Abstract

**Background:**

In this retrospective cohort study, we explored the correlation of blood human myxovirus resistance protein A (MxA) level with severity of disease in hospitalized COVID-19 patients.

**Methods:**

All 304 patients admitted for COVID-19 in our hospital until 30th of April 2021 were included in this study. MxA was measured from peripheral blood samples in 268 cases. Patients were divided into groups based on their level of MxA on admission. We studied baseline characteristics and severity of disease on admission based on clinical parameters and inflammatory biomarker levels in each group. Severity of disease during hospitalization was determined by the applied level of respiratory support, by the usage of corticosteroids and by the duration of hospitalization.

**Results:**

Higher MxA levels on admission were associated with a shorter duration of symptoms before admission, and with more severe disease. Adjusted Odds Ratios for any respiratory support were 9.92 (95%CI 2.11–46.58; p = 0.004) in patients with MxA between 400 μg/L and 799 μg/L (p = 0.004) and 20.08 (95%CI 4.51–89.44; p < 0.001) in patients with MxA ≥ 800 μg/L in comparison with patients with initial MxA < 400 μg/L. The usage of corticosteroids was significantly higher in the high-MxA group (77%) in comparison with the intermediate-MxA group (62%, p = 0.013) and low-MxA group (47%, p < 0.001).

**Conclusions:**

Higher initial levels of MxA were associated with more severe COVID-19. MxA may be a helpful additional biomarker to predict the severity of the disease.

## Background

In Finland, incidence of severe acute respiratory syndrome coronavirus 2 (SARS-CoV-2) infection was relatively low in the first year of the pandemic with 87 404 COVID-19 cases (1.6% of the population) reported by 30th of April 2021 [1]. Although several factors, including elevated levels of inflammation markers such as C-reactive protein (CRP) and interleukin-6 (IL-6) correlate with severity of COVID-19, the course of severe disease remains highly unpredictable [2].

Human myxovirus resistance protein A (MxA) is an intracellular GTPase, exclusively induced by type I and III interferons (IFNs) [3]. It inhibits production of new viruses at early stage of their life cycle by trapping nucleocapsid-like structures and making them unavailable for production [4]. It is detectable within hours of IFN stimulation and its half-life is about 2.3 days, which indicates that MxA may be a specific indicator for acute or very recent virus infection [5]. The IFN system is part of host's innate immune system against viral infections and MxA has activity against wide variety of viruses [6]. Multiple studies suggest that blood MxA can be used as a biomarker in children with respiratory virus infections [7–9]. A recent study shows that MxA could be used as a part of COVID-19 diagnostics in patients with suspected COVID-19 infection. [10]

Severe COVID-19 is associated with dysregulation of the interferon response [11]. Rapid recognition of viral RNA by Toll-like receptors 3 and 7 and RIG-I-like receptors induces a type I IFN response that may suppress viral replication, whereas activation of the cGAS-STING pathway by macrophages as a result from endothelial cell damage induces a prolonged elevation of type I IFNs which may induce aberrant inflammation and is associated with poor clinical outcome [12–14]. Inappropriate IFN responses, either caused by autoantibodies against IFNs or by inborn genetic errors, were detected in a substantial selection of patients with life-threatening COVID-19, whereas these errors are rare in the general population. [15, 16]

In this retrospective cohort study, we explored the association of blood MxA levels with severity of disease in hospitalized COVID-19 patients.

## Methods

All COVID-19 patients admitted to Turku University Hospital until 30th of April 2021 were included in this study. First admission was on March 6th, 2020. All patients were tested positive for SARS-CoV-2 by polymerase chain reaction (PCR). Patients were divided into three groups based on the blood MxA level on admission: low-MxA group included patients with MxA < 400 μg/L, intermediate-MxA group included patients with MxA 400—799 μg/L and high-MxA group included patients with MxA of 800 μg/L or higher.

Baseline clinical findings were documented as the first clinical measurements made on admission. Baseline laboratory tests, such as MxA, IgG antibodies, IL-6, CRP, and other inflammatory markers, were performed on the first weekday upon admission as part of routine care on COVID-19 departments of our hospital. Native peripheral oxygen saturation was measured before starting supplemental oxygen. CRP, IL-6, procalcitonin (PCT), ferritin and blood lymphocytes were analyzed using standard methods. Whole blood MxA protein was measured by enzyme immunoassay as earlier described and its basal level in healthy adult population is < 50 μg/L [17]. The range of detection of the assay used in this study was 10–800 μg/L. Severity of disease during admission was determined by oxygen saturation levels and body temperature on admission, level of respiratory support needed, usage of corticosteroids, and median duration of hospitalization.

SARS-CoV-2 immunoglobulin G (IgG) antibodies were analyzed using commercial automated test workstations (Liaison, DiaSorin, Italy; Alinity i, Abbott) in the Clinical Microbiology laboratory. Liaison SARS-CoV-2 test is a chemiluminescence immunoassay and detects IgG antibodies against SARS-CoV-2 surface S1 and S2 proteins. Alinity i is a two-step chemiluminescent microparticle immunoassay for detecting IgG antibodies against virus nucleocapsid protein. Presence of IgG antibodies both to virus surface protein as well as to nucleocapsid protein were measured from all samples.

Descriptive statistics were used to characterize patients and compare groups based on MxA levels on admission. Frequencies and percentages (%) were used for categorical variables, medians as well as lower (Q1) and upper (Q3) quartiles for continuous variables. Trends of continuous variables in the three MxA groups were evaluated with the Jonckheere-Terpstra test and Pearson’s chi-squared test was used for categorical data. Pairwise comparisons after Pearson’s chi-squared test were made running the same test again for two groups at a time. Binary logistic regression was performed where application of any respiratory support was a dependent variable and MxA category, age, body mass index (BMI), baseline CRP level, presence of SARS-CoV-2 specific IgG, and duration of symptoms before MxA measurement (as categorized ≤ 9 days versus > 9 days), were predictive variables. For this purpose, short duration of symptoms was defined as ≤ 9 days and long duration of symptoms as > 9 days based on median duration of symptoms in the whole patient group. Adjusted odds ratios (OR) are reported together with 95% confidence intervals (95%CI). P-values less than 0.05 (two-tailed) were considered as statistically significant. Statistical tests were performed using SAS software, Version 9.4 of the SAS System for Windows (SAS Institute Inc., Cary, NC, USA) and IBM SPSS Statistics 27 for Windows and/or Mac.

## Results

### Baseline characteristics

304 COVID-19 patients had been admitted to Turku University Hospital by 30th of April 2021. Of the patients, 139 (46%) were female, median age was 58 years (range 0–94 years) and median BMI was 29.8 kg/m^2^. Median duration of hospital stay was 7 days; 228 patients (75%) were discharged home, 63 patients (21%) were referred to another hospital or health center, and 13 patients (4%) died in study hospital. 57 patients (19%) were admitted to intensive care unit (ICU) and in those, median duration of ICU stay was 9 days.

MxA level was measured in 268 patients (88%). Patients were divided into groups based on their MxA level on admission. 36 patients (13%) had low levels (< 400 μg/L), while 73 patients (27%) had intermediate levels (400 μg/L—799 μg/L) and 159 patients (59%) had high levels of MxA (at least 800 μg/L). Age, BMI, smoking status, vaccination status for COVID-19, common comorbidities, and home medication were similar between the groups (Table 1).

**Table 1:** Baseline characteristics on admission to the hospital of patients with low MxA, intermediate MxA and high MxA

	Low MxA < 400 μg/L (N = 36)	Intermediate MxA 400–799 μg/L (N = 73)	High MxA ≥ 800 μg/L (N = 159)	p-value
Female gender, n/N (%)	18/36 (50%)	31/73 (43%)	66/159 (42%)	0.647^^^
Age (years)	61	58	58	0.874^#^
Q1–Q3	48–71	44–69	48–69	
BMI (kg/m^2^)	27.1	30.1	29.9	0.237^#^
Q1–Q3	24.0–31.8	26.1–34.6	27.0–34.5	
Smoking status, n/N (%)				0.306^^^
Non-smoker	16/26 (62%)	39/64 (61%)	71/133 (53%)	
Ex-smoker	7/26 (27%)	20/64 (31%)	56/133 (42%)	
Smoker	3/26 (12%)	5/64 (8%)	6/133 (5%)	
Vaccination for COVID-19				0.563^^^
Unvaccinated	36/36 (100%)	71/73 (97%)	154/159 (97%)	
Partially vaccinated	0/36 (0%)	2/73 (3%)	5/159 (3%)	
SARS-CoV-2-specific IgG (%), n/N (%)	24/30 (80%)	30/61 (49%)	61/138 (44%)	0.002^^^
Native SpO_2_ (%)	96	95	94	< 0.001^#^
Q1–Q3	95–98	92–96	89–96	
Temperature (°C)	37.5	37.9	38.2	0.012#
Q1–Q3	37.2–38.4	37.4–38.5	37.6–38.7	
Blood lymphocytes (× 10^9^/L)	1.23	0.99	1.05	0.583#
Q1–Q3	0.70–1.53	0.72–1.21	0.71–1.31	
CRP (mg/L)	40	48	64	0.128^#^
Q1–Q3	14–109	17–107	36–106	
PCT (μg/L)	0.09	0.12	0.14	0.017^#^
Q1–Q3	0.05–0.19	0.65–0.24	0.07–0.32	
Interleukin-6 (ng/L)	11.3	18.7	36.9	< 0.001^#^
Q1–Q3	3.9–55.4	7.2–54.1	15.7–80.3	
Ferritin (μg/L)	573	678	629	0.354^#^
Q1–Q3	243–838	257–1225	337–1037	
Pre-existent comorbidities, n/N (%)				
Obesity	13/29 (45%)	29/58 (50%)	73/147 (50%)	0.883^^^
Hypertension	11/36 (31%)	27/73 (37%)	59/159 (37%)	0.751^^^
Diabetes Mellitus	6/36 (17%)	15/73 (21%)	43/159 (27%)	0.309^^^
Obstructive Sleep Apnea	5/36 (14%)	9/73 (12%)	28/159 (18%)	0.561^^^
Asthma	5/36 (14%)	8/73 (11%)	19/159 (12%)	0.906^^^
COPD	0/36 (0%)	4/73 (6%)	11/159 (7%)	0.264^^^
Home medication, n/N (%)				
Immunosuppressive	2/36 (6%)	2/73 (3%)	18/156 (12%)	0.065^
Biological	1/35 (3%)	2/73 (3%)	2/156 (1%)	0.680^
Cytostatic	0/35 (0%)	0/73 (0%)	2/156 (1%)	0.498^

^#^Jonckheere-Terpstra test; ^Pearson's chi-squared test

Values are medians for continuous variables and percentages for frequencies

*BMI* body mass index, *IgG* immunoglobulin G, *CRP* C-reactive protein, *PCT* procalcitonin, *COPD* chronic obstructive pulmonary disease, *MxA* human myxovirus resistance protein A, *Q1* lower quartile (25th percentile), *Q3* upper quartile (75th percentile), *SpO*_*2*_ oxygen saturation on admission on ambient air

### Association of MxA level with clinical presentation on admission

**Fig. 1:**
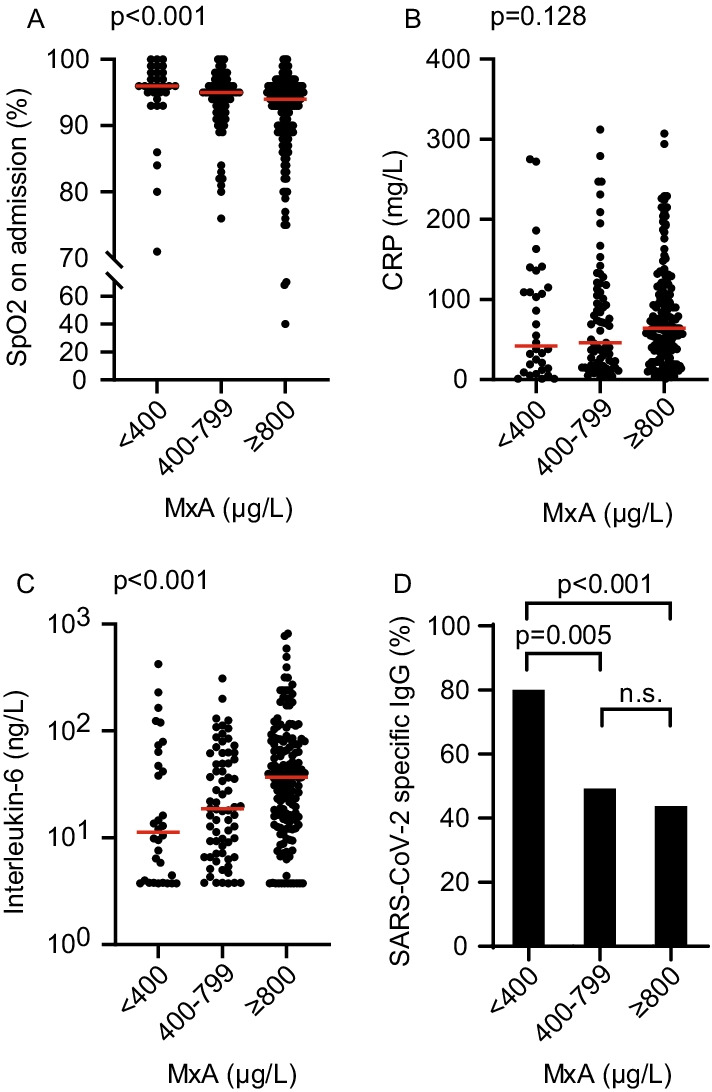
Baseline characteristics according to initial levels of human myxovirus resistance protein A. **A**–**C** SpO_2,_ CRP, and IL-6 on admission in patients with low (< 400 μg/L), intermediate (400–799 μg/L) or high (≥ 800 μg/L) initial MxA. Statistical significance was tested with the Jonckheere-Terpstra test. **D** SARS-CoV-2 IgG antibody status on admission in patients with low, intermediate and high initial MxA. Pairwise comparison was performed with Pearson’s chi-squared test. *CRP* C-reactive protein, *IgG* immunoglobulin G, *MxA* human myxovirus resistance protein A, *n.s.* not significant, *SpO*_*2*_ oxygen saturation on admission on ambient air

Higher MxA levels were associated with lower native oxygen saturation and higher body temperature upon presentation at the emergency unit. Median native oxygen saturation was 96% in the low-MxA group, 95% in the intermediate-MxA group and 94% in the high-MxA group (p < 0.001; Fig. 1A). Median body temperature was 37.5 °C in the low-MxA group, 37.9 °C in the intermediate-MxA group and 38.2 °C in the high-MxA group (p = 0.012). Higher MxA levels were associated with higher levels of inflammation markers PCT and IL-6. Median IL-6 was 11.3 ng/L in the low-MxA group, 18.7 ng/L in the intermediate MxA-group and 36.9 ng/L in the high-MxA group (p < 0.001; Fig. 1C). Median level of PCT was 0.09 μg/L in the low-MxA group, 0.12 μg/L in the intermediate-MxA group and 0.14 μg/L in the high-MxA group (p = 0.017).

Other laboratory markers of inflammation, such as lymphocyte count, CRP (Fig. 1B) or ferritin levels, were similar among the groups. Furthermore, higher MxA levels were associated with a shorter median duration of symptoms before MxA measurement (low-MxA group: 11 days; intermediate MxA-group: 10 days and high-MxA group: 8.5 days, p < 0.001).

IgG antibodies against SARS-CoV-2 could be detected in 80% of patients in the low-MxA group, in 49% of patients in the intermediate-MxA group and in 44% of patients in the high-MxA group (low- versus intermediate-MxA group p = 0.005; low- versus high-MxA group p < 0.001; Fig. 1D).

Baseline clinical parameters are presented in Table 1 and Fig. 1.

### Association of MxA level with severity of disease during admission

**Fig. 2:**
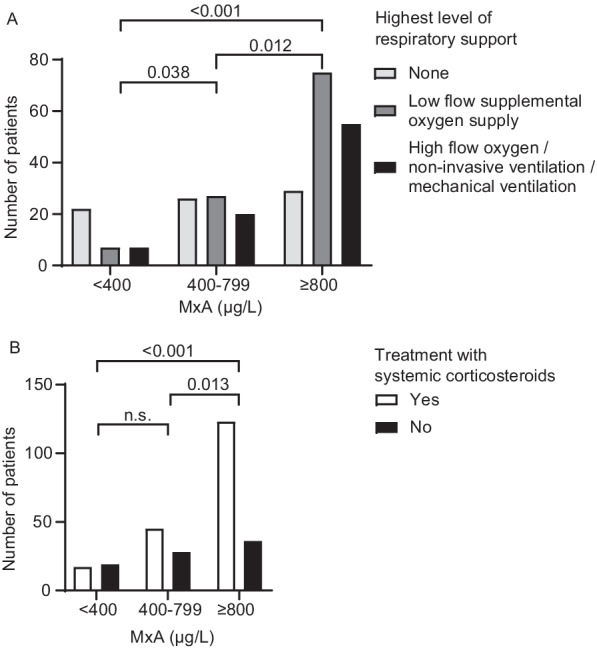
Treatment outcome according to initial levels of human myxovirus resistance protein A. **A** Highest level of respiratory support in patients with low (< 400 μg/L), intermediate (400–799 μg/L) or high (≥ 800 μg/L) initial MxA. **B** Systemic corticosteroid treatment applied in patients with low, intermediate and high initial MxA. Statistical significance was tested with Pearson’s chi-squared test. *MxA* human myxovirus resistance protein A; *n.s.* not significant

The need for any respiratory support, defined as supplemental oxygen and/or high flow nasal cannula, non-invasive ventilation or invasive mechanical ventilation, was significantly higher in the high-MxA group (82%) in comparison with the intermediate-MxA group (64%, p = 0.012) or low-MxA group (39%, p < 0.001) as well as in the intermediate-MxA group in comparison with the low-MxA group (p = 0.038), as depicted in Table 2 and Fig. 2A.

**Table 2:** Treatment categories and in-hospital mortality in patients with different MxA levels

	Low-MxA < 400 μg/L (N = 36)	Intermediate MxA 400–799 μg/L (N = 73)	High MxA ≥ 800 μg/L (N = 159)	p-value
Respiratory support, n/N (%)				< 0.001
No respiratory support	22/36 (61%)	26/73 (36%)	28/158 (18%)	
Supplemental oxygen (≤ 15 L/min)	7/36 (19%)	27/73 (37%)	75/158 (48%)	
HFNC/NIV/Intubation	7/36 (19%)	20/73 (27%)	55/158 (35%)	
ICU, n/N (%)				0.329
No	28/36 (78%)	53/73 (73%)	101/159 (64%)	
Yes	4/36 (11%)	14/73 (19%)	35/159 (22%)	
DNR	4/36 (11%)	6/73 (8%)	23/159 (15%)	
Corticosteroids, n/N (%)	17/36 (47%)	45/73 (62%)	123/159 (77%)	< 0.001
In hospital mortality, n/N (%)	1/36 (3%)	2/73 (3%)	10/159 (6%)	0.416

*HFNC* high-flow nasal cannula, *NIV* non-invasive ventilation, *ICU* intensive care unit, *DNR* do-not-resuscitate, *MxA* human myxovirus resistance protein A

P-values of frequencies were tested with Pearson's chi-squared test

Binary logistic regression was used to calculate adjusted OR for any respiratory support with baseline level of MxA, age, BMI, baseline CRP value, detection of SARS-CoV-2 specific IgG antibodies at baseline, and duration of symptoms before MxA measurement. Adjusted OR for any respiratory support was 9.92 (95%CI 2.11–46.58) in the intermediate-MxA group (p = 0.004) and 20.08 (95%CI 4.51–89.44) in the high-MxA group (p < 0.001) in comparison with the low-MxA group. Adjusted OR for any respiratory support also increased significantly with age (adjusted OR 1.03 per year, 95%CI 1.00–1.06, p = 0.041) and with baseline CRP (adjusted OR 1.02 per unit mg/L, 95%CI 1.01–1.03, p = 0.001), while BMI, duration of symptoms and detectable SARS-CoV-2 specific IgG antibodies were not associated significantly with adjusted OR’s. Results from binary logistic regression are presented in Table 3.

**Table 3:** Adjusted odds ratios for any respiratory support by binary logistic regression

	Adjusted odds ratio	95% Confidence interval	p-value
Low MxA (< 400 μg/L)	–	–	**-**
Intermediate MxA (400–799 μg/L)	9.92	2.11–46.58	0.004
High MxA (≥ 800 μg/L)	20.08	4.51–89.44	< 0.001
Age; per year	1.03	1.00–1.06	0.041
BMI; per kg/m^2^	1.02	0.96–1.10	0.474
CRP; per unit mg/L	1.02	1.01–1.03	0.001
SARS-CoV-2 IgG antibodies	1.18	0.41–3.36	0.758
Duration of symptoms ≤ 9 days	–	–	–
Duration of symptoms > 9 days	1.06	0.94–1.20	0.360

*BMI* body mass index, *CRP* C-reactive protein, *IgG* immunoglobulin G, *MxA* human myxovirus resistance protein A

The usage of corticosteroids was significantly higher in the high-MxA group (77% of patients) in comparison with the intermediate-MxA group (62%, p = 0.013) and low-MxA group (47%, p < 0.001), as depicted in Table 2 and Fig. 2B.

Antibiotics were administered in 10 patients in the low-MXA group (28%), 27 patients in the intermediate-MxA group (37%) and 40 patients in the high-MxA group (25%, p = 0.179), usually without evidence of bacterial co-infection. Bacterial co-infections were documented in only 6 patients (2%), of which 5 were in the high-MxA group and 1 in the low-MxA group (p = 0.381), while viral, fungal or parasitic coinfections were documented in none.

Higher MxA levels were associated with longer median duration of hospitalization (low-MxA group: 6 days; intermediate-MxA group: 7 days and high-MxA group: 8 days, p < 0.001). Four patients (11%) in the low-MxA group, 14 patients (19%) in the intermediate-MxA group and 35 patients (22%) in the high-MxA group were admitted to ICU (p = 0.329). One patient (3%) in the low-MxA group, 2 patients (3%) in the intermediate-MxA group and 10 patients (6%) in the high-MxA group died in the hospital (p = 0.416). The association between MxA levels and treatment categories are displayed in Table 2, Table 3 and Fig. 2.

## Discussion

In our study, high levels of initial MxA were associated with severe disease in patients admitted for COVID-19 as depicted by higher level of IL-6 and lower initial oxygen saturation at presentation, and a higher need for respiratory support and for treatment with corticosteroids during hospitalization. This is in line with findings of Mataki et al. in a small group of 45 patients [18]. In addition, there was a significantly shorter time between onset of symptoms and presentation in patients with high MxA level, and a lower chance to have detectable IgG antibodies against SARS-CoV-2, which might indicate that these patients had a quicker deterioration in early inflammatory phase of COVID-19.

MxA is nearly exclusively induced by type I and III IFNs. Several researchers have proposed models of stages of COVID-19 with interferon response prior to severe stage of the disease in which lung damage occur [13, 19]. Other studies have shown that imbalanced response of type I and III IFNs in patients with COVID-19 was associated with more severe disease [20, 21]. MxA is a robust surrogate marker of IFN responses, but any other role of MxA in the severity of COVID-19 remains unclear.

MxA is typically high in the early phase of viral infection. High MxA levels may indicate a stronger type I or type III IFN response and subsequently enhanced collateral damage from an imbalanced immune response. Furthermore, it can be hypothesized that a high level of MxA reflects a higher viral load, which by itself is associated with poor outcome in COVID-19 [22, 23]. Viral load correlates with T cell activation in several chronic viral infections [24]. Therefore, a likewise correlation between COVID-19 and markers of naive immunity would be worth exploring. A prospective study investigating serial MxA levels in combination with viral loads in early infection could clarify these issues. Furthermore, we propose that also in other viral infections, MxA levels could be related to the duration of disease in order to explore the factor of time in immune responses and disease.

Markedly elevated MxA is specific for viral infections, but MxA may also be slightly elevated in bacterial infections. [25] In our cohort, co-infections were documented in only 2% of patients, while 29% of patients received antibiotics. Even though non-therapeutic interventions were associated with a marked decrease in incidence of many common bacterial and viral pathogens during the study period [26]; we cannot rule out that in some cases, co-infections were left undiagnosed. For instance, PCR-facilities for other infections were compromised during the study period due to a shortage of reagents. Due to the low number of coinfections, our study provides little insight on the interplay between co-infections and MxA.

Our study has several limitations. First, due to the retrospective design of our study, documentation was not complete on all variables. Second, this study included only patients hospitalized in a university hospital, which may cause a bias towards severe disease and younger patients, as mild cases are treated at home and fragile patients with severe disease may receive palliative care in a local health center or at home. Third, a substantial proportion of patients had MxA levels above the upper limit of detection, which urged us to categorize patients into low-, intermediate- and high-MxA groups. Exact values might have contributed to additional understanding of associations between MxA levels and clinical outcome. Fourth, data after hospitalization was not available in most cases, which may lead to underreporting of complications.

Dynamics of the pandemic should be considered when interpreting our findings. The proportion of vaccinated people in Finland remained relatively low until the data cut off, leaving all our patients without full vaccination for COVID-19. Therefore, effects of vaccination could not be studied in this cohort. Furthermore, different variant strains of SARS-CoV-2 may account for different degrees of interferon responses and different courses of disease.

## Conclusions

In our retrospective cohort of patients admitted with COVID-19, higher initial level of MxA was associated with more severe COVID-19, with increased treatment intensity and with prolonged hospital admission. Therefore, we consider that blood MxA level may serve as an additional biomarker in COVID-19.

## Data Availability

The datasets generated and analysed during the current study are not publicly available due to privacy of study objects but are available from the corresponding author on reasonable request.

## Abbreviations

BMI: Body mass index
COPD: Chronic obstructive pulmonary disease
CI: Coincidence interval
CRP: C-reactive protein
DNR: Do-not-resuscitate
HFNC: High-flow nasal cannula
ICU: Intensive care unit
IFN: Interferon
IgG: Immunoglobulin G
IL-6: Interleukin 6
MxA: Human myxovirus resistance protein A
NIV: Non-invasive ventilation
n.s.: Not significant
OR: Odd’s ratio
PCT: Procalcitonin
Q1: Lower quartile (25th percentile)
Q3: Upper quartile (75th percentile)
SpO_2_: Oxygen saturation on admission on ambient air
